# Operating cost analysis of a concentric aluminum tubes electrodes electrocoagulation reactor

**DOI:** 10.1016/j.heliyon.2019.e02307

**Published:** 2019-08-14

**Authors:** Forat Yasir AlJaberi

**Affiliations:** Chemical Engineering Department, College of Engineering, Al-Muthanna University, Al-Muthanna, Iraq

**Keywords:** Chemical engineering, Electrochemistry, Simulated wastewater, Operating cost, Statistical analysis, Electrocoagulation

## Abstract

Energy consumed by the electrochemical treatment of wastewater has more responsibility in designing such a process. The objective of the present study was to investigate the effect of electrodes geometry used in the electrocoagulation cell on the operating cost that required to remove lead from the industrial wastewater. The studied operating variables in the present study were the initial concentration of lead (10–300)ppm, electrolysis time (5–60)minutes, applied electric current (0.5–2.5)Amps., pH (2–12), and stirring speed (0–300)rpm. It has shown that cost was affected by the configuration of aluminum electrodes as well as other studied parameters. Statistical methods and programs were used to estimate an empirical correlation of the operating cost as well as the consumption of aluminum electrodes and energy.

## 1 Introduction

Natural and domestic sources of polluted water with toxic metals are annually discharged into the environment due to the continuous requirement of these metals and their components in several factories which leads to a significant threat for the environmental and human [1].

Aqueous environments are among the most remarkable eco-systems concerning chemical pollution. Toxic metal pollution has become a significant worldwide crisis, therefore essential economic aspects of efficient methods should be performed to remove toxic metals from polluted waters to ensure the availability of recover the treated wastewater according to the acceptable specifications.

The treatment of the contaminated water effluents released from different activities required effective techniques for removing toxic metal ions. (i.e. recovering of heavy metals as a result). Several methods had been used for this purpose such as precipitation, reverse osmosis, adsorption, ion exchange, chemical coagulation and electrochemical method [2, 3, 4, 5, 6]. The electrochemical technique provides removing of toxic pollutants through the reactions of oxidation and reduction with significant removal efficiency and notable energy efficiency. Whereas other techniques could not recover ions of these pollutants as metals and the other disadvantage is the large amounts of sludge released from these techniques. Electrochemical methods are simple, fast, inexpensive, easily operable and eco-friendly in nature. Besides, purified water is potable, clear, colorless and odorless with low sludge production.

Contaminant removal happens by two cooperative operations, electrocoagulation and electrofloatation processes. The former one depends on the dissolution of the anode electrode due to the formation of aluminum hydroxyl component (i.e. electro-coagulant) which works as adsorbents. While the latter process depends on the release of hydrogen gas at the cathode electrode as well as oxygen gas producing at the anode electrode where these gases bubbles take the light weight of pollutant to the surface of the simulated solution in the electrocoagulation reactor [7].

Electrocoagulation is a clean electrochemical process, which uses an applied voltage (i.e. electrical current) to remove metals from solution. This technique has the ability to eliminate the drawbacks of the conventional treatment techniques to achieve a sustainable and economic treatment of polluted industrial wastewater [3, 8]. It is accomplished according to the following three successive consecutive steps [9]:

1. Generation of flocs on the electrodes due to the redox reactions as follow:
  • At the anode electrode with metal M:

(1) M_(S)_ ⇒ M^+n^_(aq)_ + ne^−^

(2) 2H_2_O ⇒ O_2_ + 4H^+^ + 4e^−^

• At the cathode electrode:

(3) 2H_2_O + 2e^−^ ⇒ H_2(g)_ + 2OH^−^_(aq)_

2. Destabilization of the contaminants at the cathode surface as follows:

(4) M^+n^_(aq)_ + ne^−^ ⇒M_(S)_

3. Accumulation of the unsettle components to generate flocs.

Therefore, the electrocoagulation technique comprises two important processes as revealed in (Eqs. 5 and 6) as follow:

(5) 2Al ⇒ 2Al^+3^ + 6e ^-^

(6) Al^3+^ +3OH^−^ ⇔ Al(OH)_3_

There are many factors affecting the geometry of electrodes such as the shape of electrode where the present research had employed a tube shape as electrode. Moreover, the distance between the electrodes is important also where the present design of electrodes was used concentric tubes which means that parameter is fixed [9].

Since this technique required electric current in order to accomplish its process depending on releasing different ions in the electrocoagulation reactor along the period of wastewater treatment as a result of consuming electrodes, therefore, the cost required to operate such type of reactor is extremely essential. From an economic view, the total cost of operating the electrocoagulation reactor was estimated according to the following equation (Eq. 7) [10]:

(7) TOC = a × M_AEC_ + b × E_CONS_

where:

TOC: Total operating cost ($/m^3^).

a: Price of unit weight of electrode ($/mg) [ at the research time equals 9.7×10–6 $/mg].

M_AEC_: Weight of actual electrode consumed (mg/m^3^).

b: Price of unit electrical energy ($/kW.h) [ at the research time equals 0.008 $/kW.h].

E_CONS_: The energy consumed (kW.h/m^3^).

The energy consumption (kWh/m^3^) depends on the amounts of the electrical current as well as the voltage applied to the electrochemical cell. It was calculated according to the following equation (Eq. 8) [11]:

(8) E= (U. I. t)/(1000.V)

where: U is the voltage applied (volt), I: applied electric current (Amps.), t: electrolysis time (h), and V is the volume of the synthesis wastewater (m^3^).

Obviously, the amount of consumed energy will be minimized and cost-effective from the economic insight. Half-factorial of a central composite method under RSM technique will be used to design experiments via Box-Wilson technique and analysis the result by using Statistica-10 and Minitab-17.

## 2 Experimental

### 2.1 Instrumentation

**Fig. 1:**
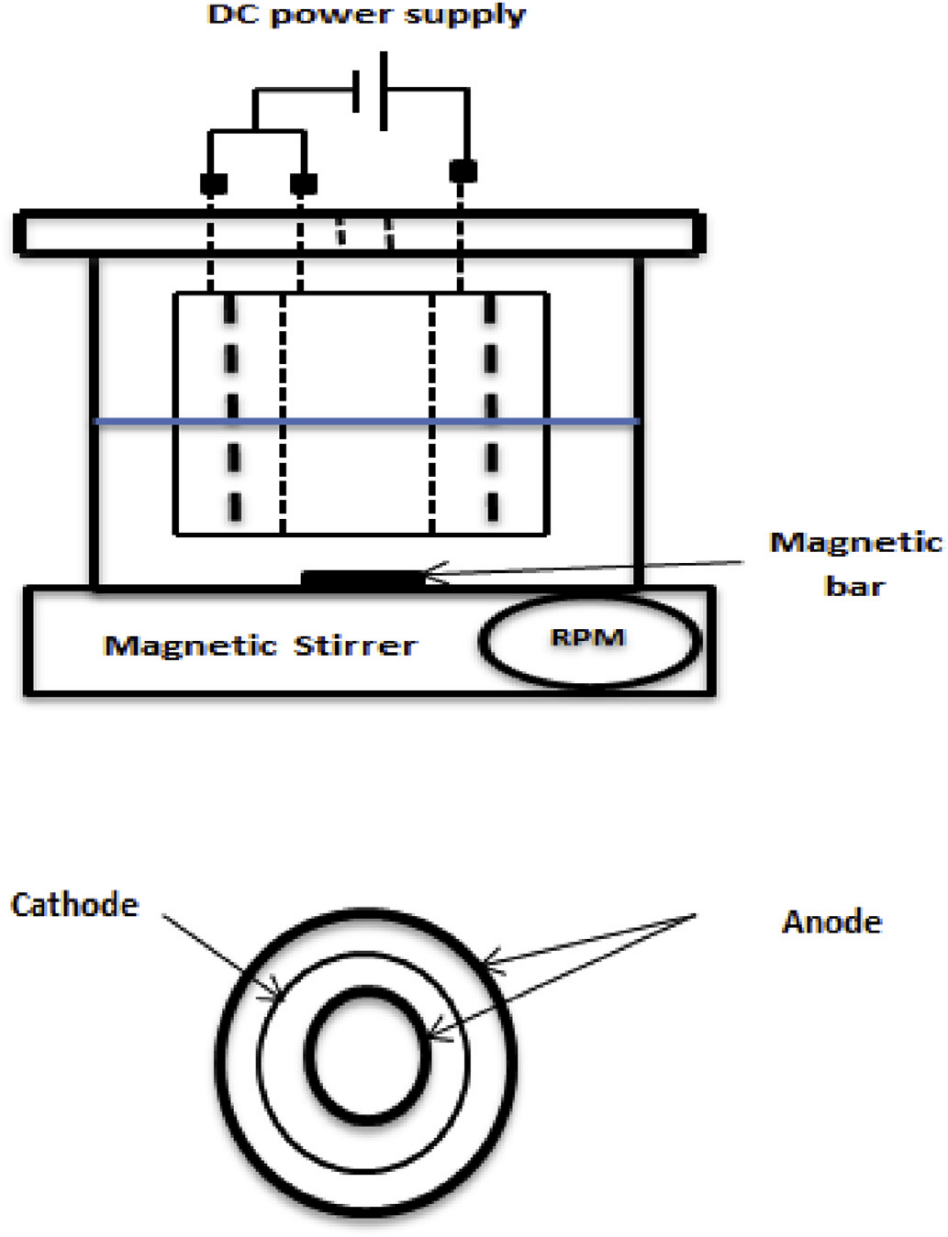
Schematic of the electrocoagulation cell and the electrodes configuration.

Electrodes constructed of triple concentric aluminum tubes with different diameters and thicknesses. A monopolar arrangement of these electrodes was employed in a batch electrocoagulation reactor as shown in Fig. 1 with an active area equals approximately 285 cm^2^. Anode electrode includes the outer and inner tubes while the mid tube was classified as the cathode electrode.

Table 1 list the description of the aluminum tubes that were used as electrodes of the electrocoagulation reactor via the treatment of the polluted water.

**Table 1:** Description of aluminum electrodes.

Tubes	Classification of electrodes	Height (cm)	Wet Height (cm)	Tube thick (cm)	Outer dia. (cm)	Inner diameter (cm)	Dist. in between (cm)
Outer	Anode	9.7	4	0.2	7.5	7.3	1.6
Mid.	Cathode	8.5	4	0.15	5.7	5.55	1.55
Inner	Anode	7.1	4	0.3	4	3.7	------

### 2.2 Materials

Samples of synthesis wastewater were prepared according to the design of the experiments by dissolving the required amount of an initial concentration of lead nitrate Pb(NO_3_)_2_ (having 99.99 of purity; B.D.H- England) weighted by using Digital balance (500 g × 0.01 g) (PROF company), in distilled water belongs to the Eqs. [8, 9]:

(9) W= V × C_i_× (M / m_A_)

Where: W is the weight of salt (grams); V: Volume of solution (liter); C_i_: Initial concentration of lead ions in solution (ppm); M: M. wt of the lead nitrate; m_A_: Atomic weight of lead.

Hydrochloric acid (0.1 N) and sodium hydroxide (0.1 N) were performed in order to adjust the value of pH which is measured using (ATC company) measurement device. In order to enhance the conductivity and to prevent the formation of an oxidation layer on the cathode, 100ppm of NaCl was added to the pretreated simulated solution.

An external power device type digital DC- power supply (SYADGONG company-305D) model; 0–30 V and 0–5 A was used to supply the designed voltage to the cell. The result samples are collected and filtered using cellulose Glass-Microfibre discs (Grade: MGC; pore diameter is 0.47 μm- MUNKTELL). Electrodes are weighed before and after each experiment then, and they are washed one time with 0.1N HCl and three times with distilled water to ensure they were cleaned well. The same procedure was repeated for the next run according to the designed details. The ranges of the operational variables are shown in Table 2.

**Table 2:** Operational parameters.

Parameters	Ranges
Electrolysis time (min)	5–60
Initial lead concentration (ppm)	10–300
pH	2–12
Current or current density (Amps.)	0.5–2.5
Stirring speed (RPM)	0–300

### 2.3 Analysis

The mathematical correlations of the studied responses were obtained using a response surface methodology method via rotatable central composite design uniform (CCD) - two level factorial-half fraction. The empirical correlation could be achieved belongs to the following equation (Eq. 10) [11, 12, 13, 14]:

(10) $$Y=B_{0}+\sum_{i=1}^{q}B_{i}X_{i}+\sum_{i=1}^{q}B_{ii}X_{i}^{2}+\sum_{i}\sum_{j}B_{ij}X_{i}X_{j}+\varepsilon$$

X_1_, X_2_, to Xq denote the independent variables that are continuous and a controllable with negligible ε error; where B_o_, B_i_, to B_ij_ are called the regression coefficients which are unknown and to be estimated and ε is a random error (or residual) which is the amount of variation in Y.

Therefore, thirty-two runs are designed as cube points:16, center points in the cube:6, axial points:10, the center points in axial is none, and the rotatability α is 2. Table 3 explains the real and coded value of operational parameters. Statistica-10 and Minitab-17 are employed in order to estimate the regression coefficients and graphical analysis of the obtained outputs.

**Table 3:** Real and coded operational variables.

Real Variable (Xi)	Coded Variables				
	-2	-1	0	1	2
X_1_ = Electrolysis time (min.)	5	19	33	46	60
X_2_ = Lead concenration (ppm)	10	83	155	228	300
X_3_ = pH	2	5	7	10	12
X_4_ = Current (Amps.)	0.5	1	1.5	2	2.5
X_5_ = Stirring speed (rpm)	0	75	150	225	300

## 3 Results and discussion

Statistical design of experiments was executed to investigate the effect of electrodes geometry and the operating variables on the operating cost. The novel design used in the electrocoagulation reactor was innovated by the researcher, therefore, the current results are specialized for this novel design [8]. Table 4 shows the values of total operating cost (TOC) and energy consumed (E_CONS._) according to the equations Eq. 7 and Eq. 8 as well as the values of the actual electrodes consumed (M_AEC_) according to Eq. (11).

**Table 4:** Results of responses.

Run No.	M_AEC_ (mg/m^3^)	E_CONS_ (kWh/cm^2^)	Total operating cost ($/m^3^)	Run No.	M_AEC_ (mg/m^3^)	E_CONS_ (kWh/cm^2^)	Total operating cost ($/m^3^)
1	0.125	3.20	0.026	17	0.025	1.80	0.014
2	0.200	6.80	0.054	18	0.525	17.70	0.142
3	0.150	3.20	0.026	19	0.225	10.70	0.086
4	0.200	7.10	0.057	20	0.240	9.80	0.078
5	0.125	2.90	0.024	21	0.135	9.40	0.075
6	0.385	6.80	0.054	22	0.125	8.60	0.069
7	0.090	2.80	0.023	23	0.145	1.50	0.012
8	0.350	6.30	0.051	24	0.305	23.30	0.186
9	0.115	9.80	0.078	25	0.365	12.40	0.099
10	0.385	21.90	0.175	26	0.230	10.20	0.082
11	0.330	9.10	0.073	27	0.220	10.10	0.081
12	0.410	22.80	0.183	28	0.275	10.20	0.082
13	0.210	9.80	0.078	29	0.215	10.40	0.083
14	0.525	21.60	0.173	30	0.200	10.70	0.086
15	0.095	9.00	0.072	31	0.185	10.60	0.085
16	0.445	21.60	0.173	32	0.195	10.20	0.082

**Fig. 2:**
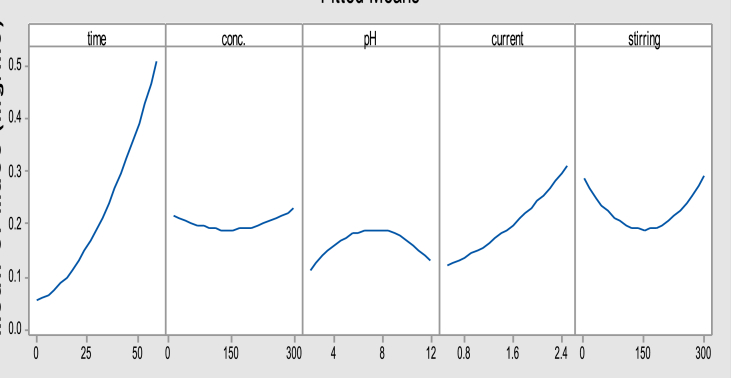
Main effects plot of variables for the actual electrodes consumption.

**Fig. 3:**
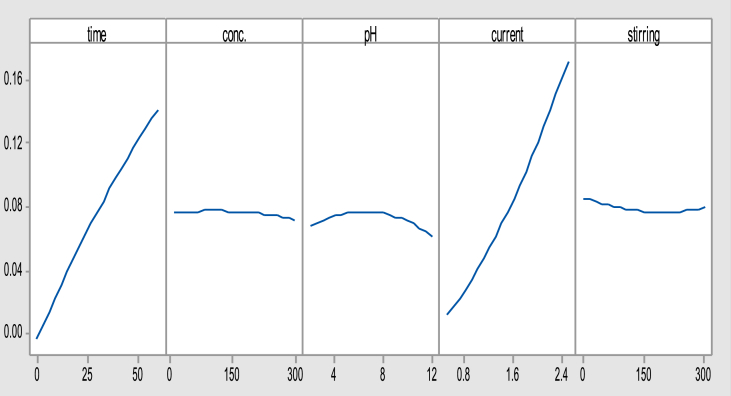
Main effects plot of variables for the total operating cost.

Figs. 2 and 3 explain, in general, the effect of the operational variables on the studied responses especially the applied current that has the main effect on this operation during the increment of electrolysis time [15, 16, 17, 18].

While other parameters are not more significantly affecting the operating cost response, they still show a notable variation on the actual electrodes consumption because the electrocoagulation process was extremely depending on the formation of coagulants in situ according to the redox reaction in order to accomplish the treatment process of the simulated wastewater [8, 19].

The mathematical correlations of the actual electrode consumption response which related to the operational parameters is described below:

Actual electrodes consumption response (*R*^*2*^*= 0.9*50):

(11) Y_MAEC_ = -0.025–0.00774 X_1_ + 0.00107 X_2_+ 0.0541 X_3_ – 0.090 X_4_ - 0.000867 X_5_ + 0.000101 X_1_^2^ + 0.000002 X_2_^2^ - 0.00275 X_3_^2^ + 0.0264 X_4_^2^ + 0.000004 X_5_^2^ - 0.000011 X_1_ X_2_+ 0.001291 X_1_ X_3_ + 0.00336 X_1_ X_4_ - 0.000021 X_1_ X_5_ - 0.000183 X_2_X_3_ + 0.000155 X_2_ X_4_ - 0.000001 X_2_ X_5_ - 0.0120 X_3_ X_4_ - 0.000043 X_3_ X_5_ + 0.000417 X_4_ X_5_

In order to find the effect of all of the operating parameters on the total operating cost response to get more information than the theoretical equation (i.e., Eq. 7), Eq. 12 explains that as follows with (*R*^*2*^*= 0.998*2):

(12) Y_TOC_ = 0.0051–0.000934 X_1_ + 0.00005 X_2_+ 0.00719 X_3_ – 0.0367 X_4_ - 0.000089 X_5_ - 0.000009 X_1_^2^ - 0.0000001 X_2_^2^ - 0.000501 X_3_^2^ + 0.01455 X_4_^2^ + 0.0000001 X_5_^2^ + 0.000001 X_1_ X_2_- 0.000023 X_1_ X_3_ + 0.002574 X_1_ X_4_ - 0.0000001 X_1_ X_5_ - 0.000005 X_2_ X_3_ - 0.000002 X_2_ X_4_ - 0.0000001 X_2_ X_5_ - 0.000108 X_3_ X_4_ + 0.000006 X_3_ X_5_ - 0.000019 X_4_ X_5_

### 3.1 Effect of electrolysis time

**Fig. 4:**
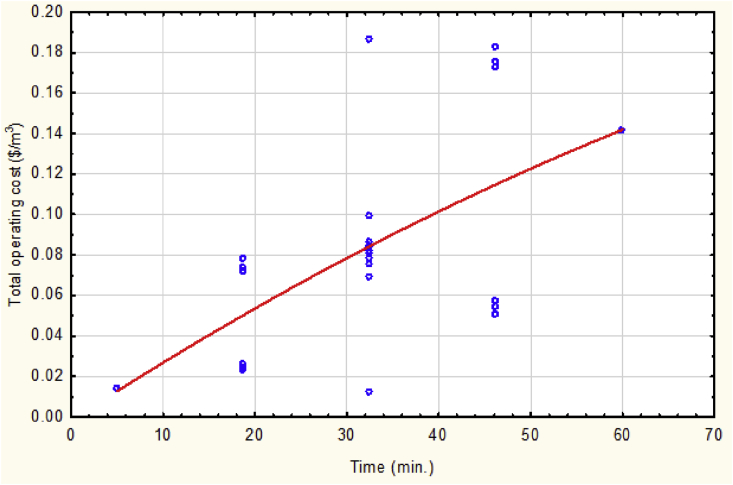
The total operating cost vs. electrolysis time at mean values of other parameters.

As shown in Fig. 4, the total operating cost was significantly affected by the time of electrolysis. Logically, this is a correct estimation due to the continuous consumption of both energy and electrodes to form the required amount of coagulants in order to accomplish the operation of wastewater treatment via the electrocoagulation reactor.

### 3.2 Effect of initial lead concentration

**Fig. 5:**
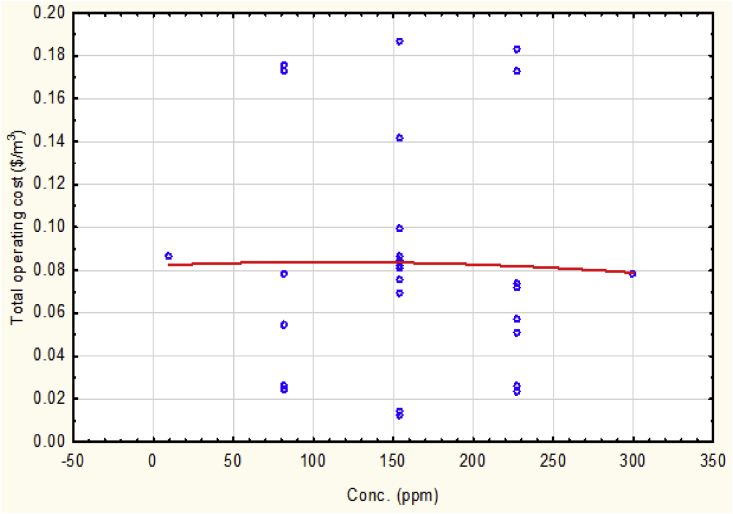
The total operating cost vs. lead concentration at mean values of other parameters.

The value of initial lead concentration has no significant impact on the operating cost value as shown clearly in Fig. 5 because of the non-direct effect between these two variables. This operational parameter may have a direct effect on the value of removal efficiency of the reactor where it may requires an enhancement of design or extended the ranges of the studied variables. The presence of lead ions increases slightly the value of solution conductivity that caused to reduce the ohmic potential drop then decreases current efficiency which is inversed proportional to the electric current supplied according to the law of Faradaic efficiency, therefore, the electric current supplied will be increased. The increment of current supplied to the reactor caused to increase the generation of coagulants, i.e. electrode consumption, and raising the consumption of energy. The increment and decrement of total operating cost varied according to the concentration of lead presented in the solution that affected the value of solution conductivity, i.e. energy consumption, and coagulants generation, i.e. electrodes consumption.

### 3.3 Effect of solution pH

**Fig. 6:**
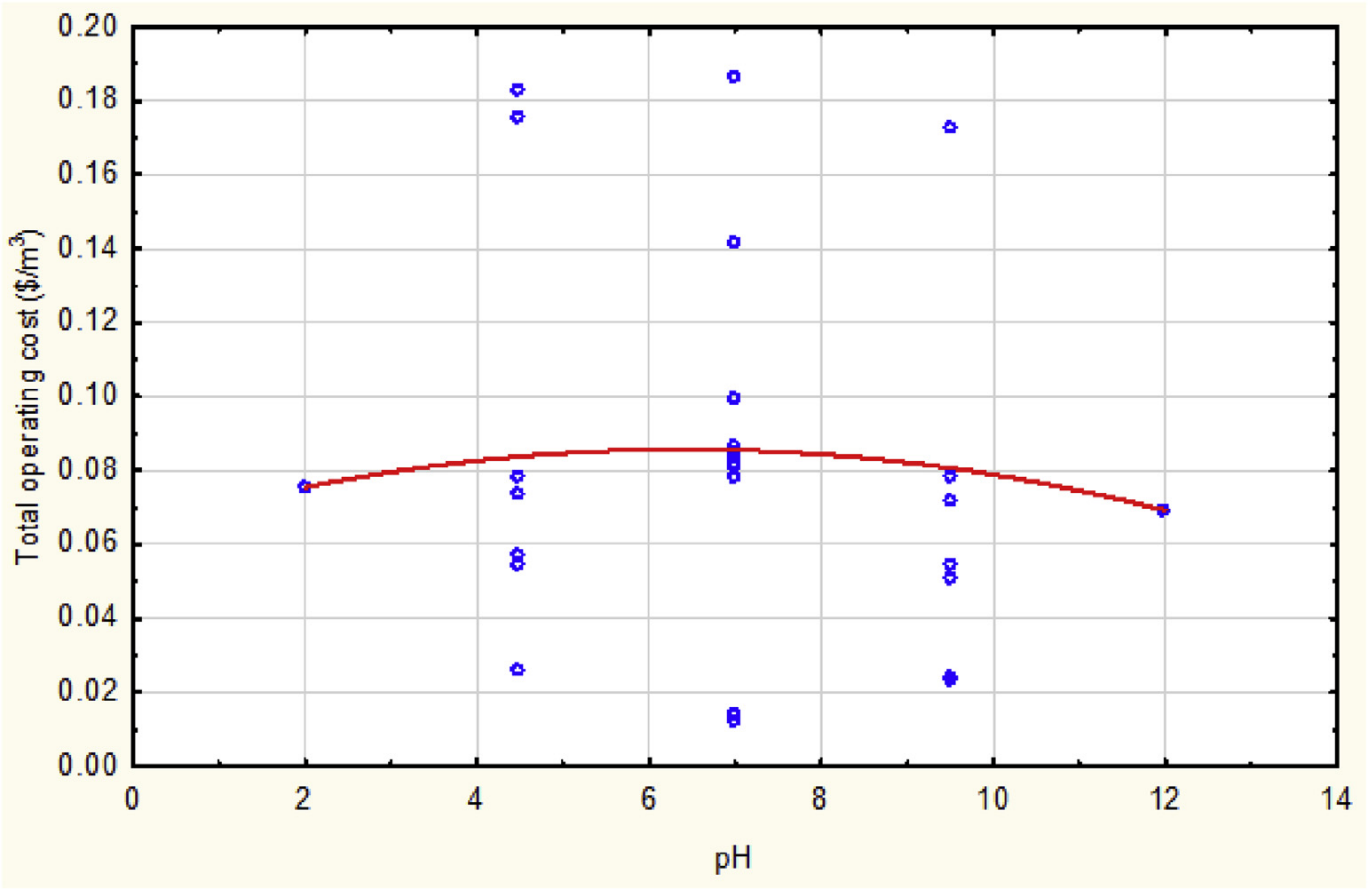
The total operating cost vs. pH at mean values of other parameters.

Fig. 6 shows an irregular behavior of operating cost along the variation of solution pH value. As the solution pH increased toward the neutral status, the total cost increases and continue like that when the treated solution be basic, but any further of increment of the alkalinity the operating cost will be minimized because the basic medium of the polluted solution possesses more of coagulants, i.e. more of electrodes consumed, that was sufficient to accomplish the adsorption process via the electrocoagulation reactor in comparison to the acidic solution, i.e. lower value of energy consumption. But the highest basic solution tends to decrease pH value when it excess 9 approximately [5, 20]. Moreover, the basic solution has more value of conductivity which caused current efficiency to be minimized and electric current raised then the consumption of electrodes and energy increased. Therefore, the behavior of the studied responses varied according to the value of solution pH.

### 3.4 Effect of current applied

**Fig. 7:**
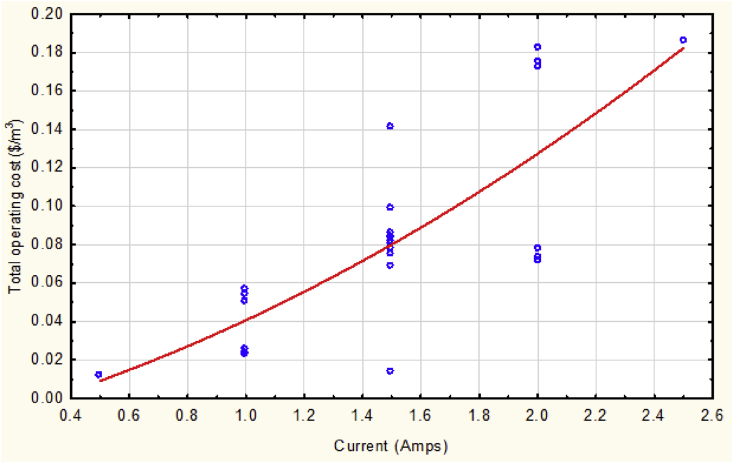
The total operating cost vs. current at mean values of other parameters.

Fig. 7 shows a direct proportional of the electric current applied to the value of total operating cost. This result seems to be logic since the main effect of such treatment process is that factor. All of the studied responses are affected by the increasing of current supplied because the specified electrodes continue in consumption as the current still applied as well as the consumption of energy [21, 22]. Therefore, the value of operating cost raises due to that consumption of both of these output responses.

### 3.5 Effect of stirring speed

**Fig. 8:**
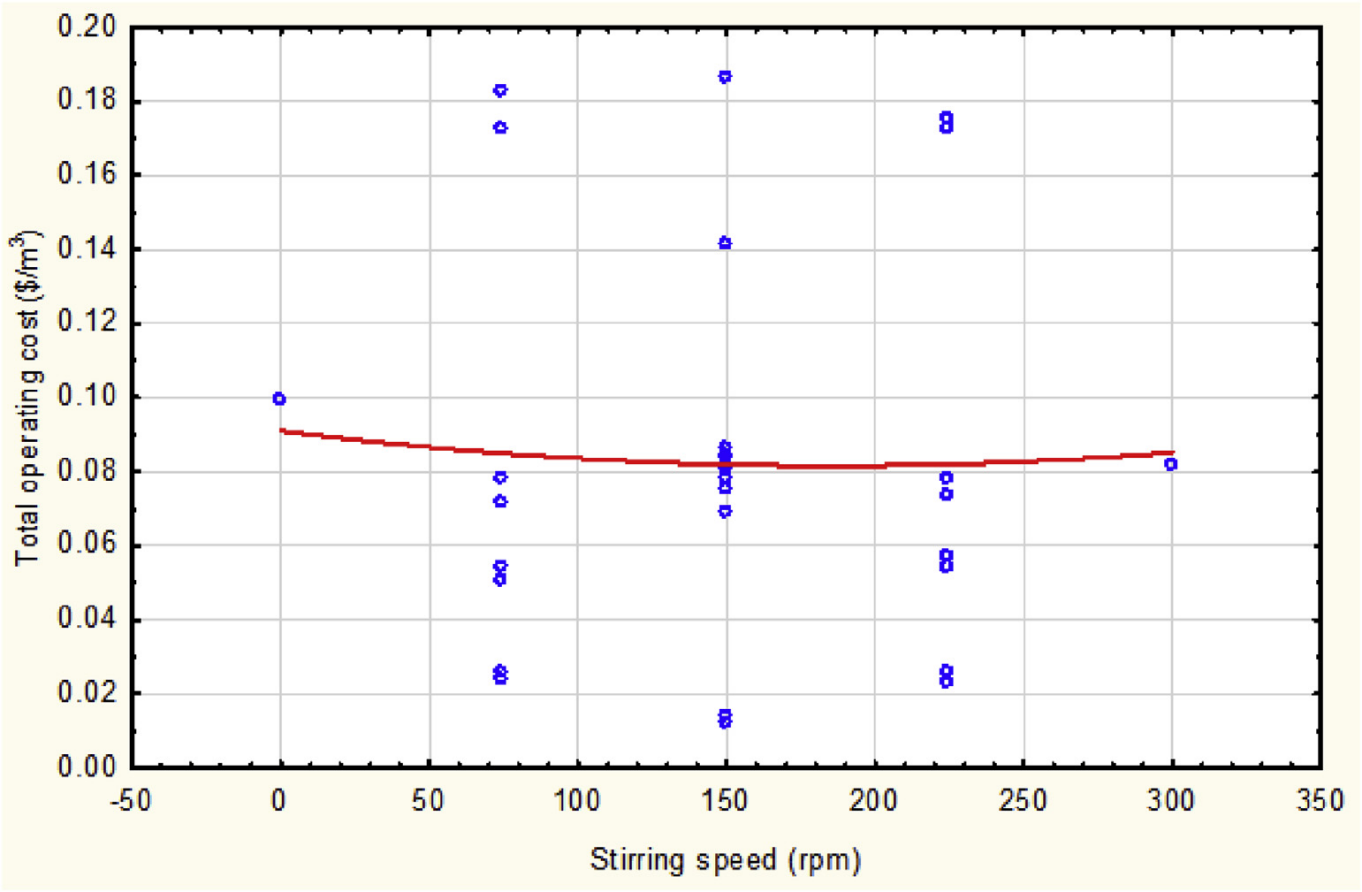
The total operating cost vs. the stirring speed at mean values of other parameters.

The behavior of the operating cost was slightly minimized with the increasing of mixing speed in then maximized but, in general, it was not more significant as shown in Fig. 8. This parameter may clearly impact other responses especially the consumption of electrodes due to the accelerating of interacting of opposite ions in the simulated wastewater that generates coagulant then adsorbed the pollutant fast.

Figs [4, 5, 6, 7, 8]. had explained the relation between the total operating cost and each one of the operational variables individually when others keeping at their mean values. The mathematical correlations of these relations are listed in Table 5.

**Table 5:** Total operating cost correlations against each one of parameters while others at mean values.

X_i_:Operational parameters	Y: Total operating cost
Electrolysis time (min.)	Y = -0.0019 + 0.003x − 9.4002E-6x^2^
Lead concenration (ppm)	Y = 0.0823 + 2.9589E-5 x − 1.3518E-7 x^2^
pH	Y = 0.064 + 0.0068x − 0.0005x^2^
Current (Amps.)	Y = − 0.0146 + 0.0397x + 0.0156x^2^
Stirring speed (rpm)	Y = 0.091 − 0.0001x + 2.7427E-7x^2^

**Fig. 9:**
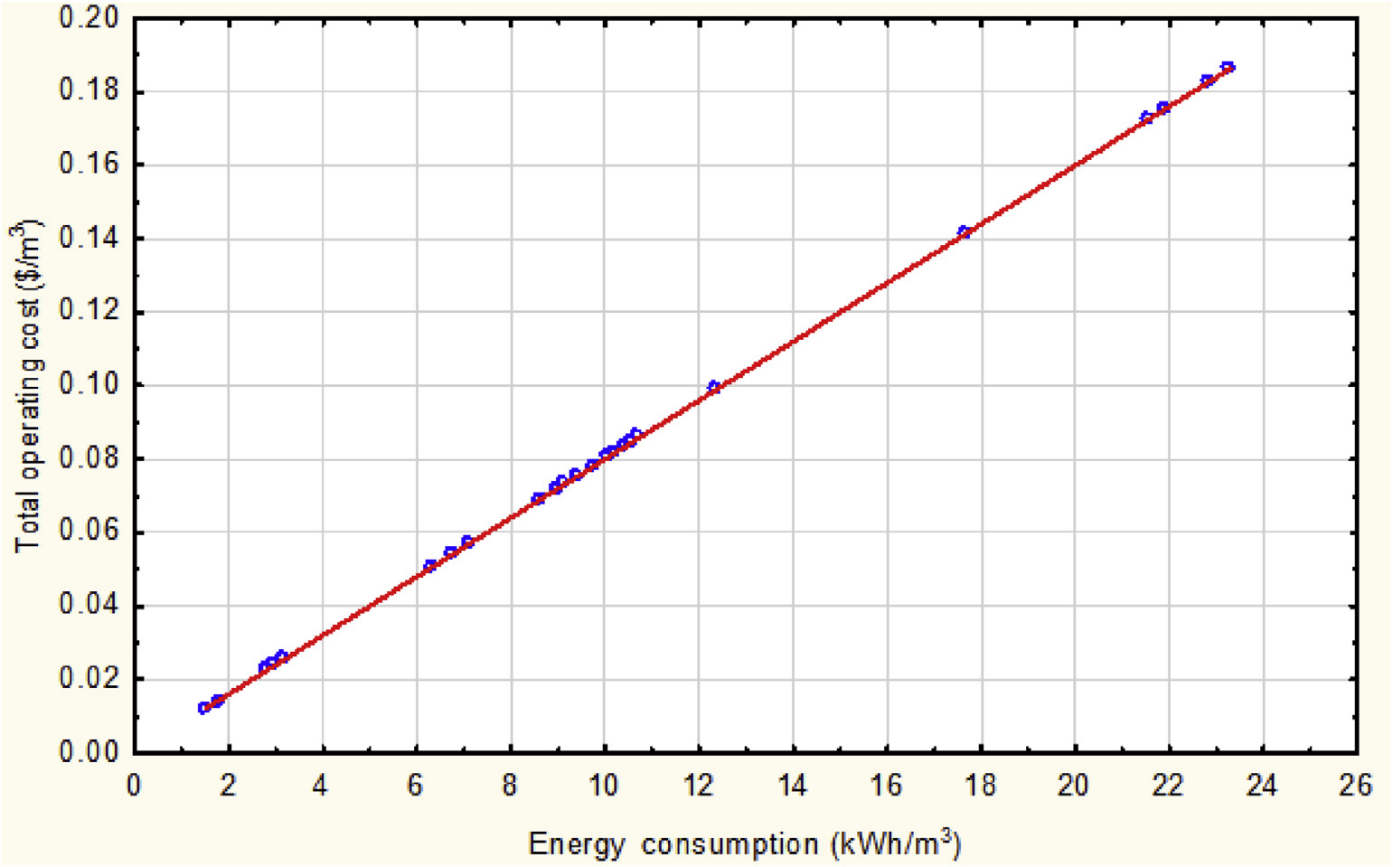
The total operating cost vs. energy consumption at mean values of the operational parameters.

Fig. 9 explains the relation between the total operating cost with the value of energy consumption according to the following empirical equation (Eq. 13):

(13) TOC = 7.289*10^−10^ + 0.008 (E_CONS_)-5.797*10^−13^ (E_CONS_)^2^

It seems to be linear since the value of the non-linearity presented in Eq. (13) was multiplied by (10E-13) which makes the linearity behavior the dominant part in that equation and its figure.

**Fig. 10:**
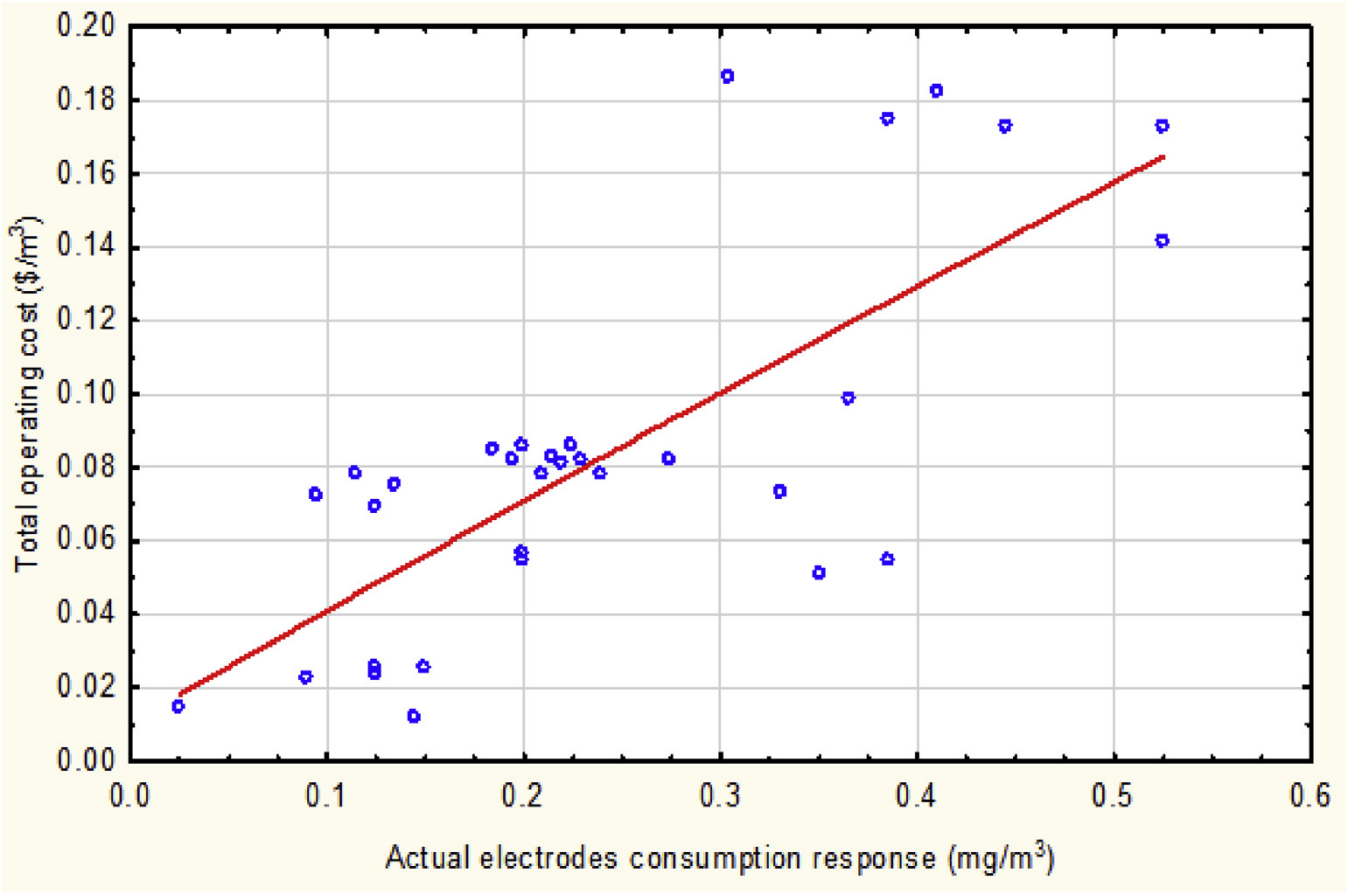
The total operating cost vs. actual electrodes consumption at mean values of the operational parameters.

Moreover, Fig. 10 relates the total operating cost to the actual electrodes consumption value according to the following equation (Eq. 14):

(14) TOC_._ = 0.0104 + 0.3083 (M_AEC_) – 0.0272 (M_AEC_)^2^

## 4 Conclusions

The present study has found the relation between the effect of electrodes geometry and their consumption as well as energy consumption in one hand and the value of operating cost on the other hand. The duration of each experiment and the electric current supplied to the novel configuration of electrodes, via the electrocoagulation treatment of wastewater, having a direct effect on the total value of the operating cost in comparison to the other variables that caused the behavior of responses to be in irregular manner due to the present of different operations occurred throughout the reactor such the redox reactions, coagulants generation and adsorption. Several of empirical mathematical correlations are estimated which give the operator the ability to monitor the requirements cost from economic view when this kind of treatment method is practically scaling up.

## Declarations

### Author contribution statement

Forat Yasir AlJaberi: Conceived and designed the experiments; Performed the experiments; Analyzed and interpreted the data; Contributed reagents, materials, analysis tools or data; Wrote the paper.

### Funding statement

This research did not receive any specific grant from funding agencies in the public, commercial, or not-for-profit sectors.

### Competing interest statement

The authors declare no conflict of interest.

### Additional information

No additional information is available for this paper.
